# Outcome-Based Self-Directed Learning (OBSDL) in Pharmacology in the Indian Context: A Convergent Mixed-Method Study

**DOI:** 10.7759/cureus.103881

**Published:** 2026-02-18

**Authors:** Shalini Chandra, Anju Saxena, Pooja Agrawal, Tripti Waghmare, Sujata Singh, Iram Shaifali

**Affiliations:** 1 Pharmacology, Rohilkhand Medical College and Hospital, Bareilly International University, Bareilly, IND; 2 Physiology, Datta Meghe Institute of Medical Sciences (Deemed to be University), Wardha, IND

**Keywords:** india, mixed-method, outcome, pharmacology, self-directed learning

## Abstract

Background: The outcome-based self-directed learning (OBSDL) allows students to achieve course outcomes (COs) in pharmacology. We aimed to implement and enhance the ability of self-directed learning (SDL) among medical undergraduates. SDL can appear in the classroom. By leveraging OBSDL as an extension to SDL, we can create a more meaningful learning experience for students that will last beyond the regurgitation of memorized content. The alignment of the curricular framework, implementation of lesson plans, sharing of SLOs, guidance for resources, and robust assessment all contribute to the achievement of OBSDL. A combination of teaching methodologies should be practiced and complemented to facilitate learning among students in competency-based medical education (CBME).

Objectives: This study aimed to implement SDL sessions to align the curricular framework with selected pharmacology topics among second-phase Bachelor of Medicine and Bachelor of Surgery (MBBS) students.

Methods: The nominal testing method was used to select competencies to be taught as SDL. The competencies selected were skeletal muscle relaxants (SMR), pharmacotherapy of glaucoma (PG), communication skills (CS), and management of common poisoning (MCP). SDL modules were prepared by the content experts. Kemp’s instructional model was used to design the lesson plan. Validation of the lesson plan was done by the Delphi method by subject experts. Kendall's coefficient of concordance test (W)and Fleiss' kappa (K)were analyzed to indicate the consensus reached by experts. Varied teaching strategies were adopted for each SDL session depending upon the nature of the competency. Thematic analysis was conducted by a two-person analysis team. Reliability and validity of thematic analysis were done by calculating intercoder reliability statistics (Cohen’s kappa) to assess the level of agreement between two coders. Robust assessments as per Bloom’s taxonomy were conducted to achieve COs. Multiple-choice questions (MCQs) were framed as per Bloom’s taxonomy, and objective structured practical examinations (OSPEs) were designed for assessment. Coding and thematic analysis were conducted for qualitative analysis.

Results: Rank concordance (W) among evaluators was strong to unusually strong (0.443, 0.450, 0.445, and 0.532; p < 0.001), and the interrater agreement (K) was fair to moderate (0.370, 0.378, 0.372, and 0.435; p < 0.001) for lesson plans of SMR, PG, CS, and MCP, respectively. The outcome for each specific competency was achieved, with a significant increase in posttest scores after SDL sessions (p < 0.001 for all sessions). Cohen's kappa was 0.672, which indicated substantial agreement between the two raters for themes. Self-learning skills, metacognition, collaboration, and autodidactism were major themes that emerged from the thematic analysis.

Conclusion: The OBSDL effectively enhances learning outcomes in the field of pharmacology. Various SDL skills, like self-learning skills, metacognition, collaboration, autodidactism, application, and experiential learning, have emerged from thematic analysis, which are essential for lifelong learning. The qualitative method has added understanding of students' perspectives. A judicious placement of various teaching-learning methods designed based on the complexity of the topic may offer a comprehensive outcome-based approach within the Indian CBME.

## Introduction

Pharmacology is frequently viewed as a challenging area of study for medical undergraduates and is frequently regarded as a challenging subject to learn. Teaching pharmacology demands a variety of methods that include interactive lectures, tutorials, small-group discussions, demonstrations, case scenario-based learning, flipped classroom sessions, and self-directed learning (SDL), and it is difficult to impart the knowledge of skills like prescription writing and adverse reporting, which are required in learning pharmacology, by using only traditional teaching methods. Traditional teaching methods do not adequately prepare students to comprehend the subject's concepts and rational therapies. Undergraduates lack the necessary skills to apply pharmacology principles in later stages of the medical curriculum [1]. It demands a great effort and planning from both teachers and students in order to achieve course outcomes (COs) [2]. In the discipline of pharmacology, where theoretical knowledge must be seamlessly integrated with rational drug use and patient care, the attainment of COs is pivotal to ensure competent and safe medical practice [3]. The National Medical Council (NMC), India, has emphasized competency-based medical education (CBME), where the alignment of competencies with teaching-learning methods and assessment strategies is a must to achieve outcomes [4, 5].

The pharmacology education is constantly evolving and is in a phase of rapid development [6]. Lifelong learning habits allow students to keep learning throughout their careers and are crucial for updating themselves with the fast-growing medical knowledge. SDL is one of the approaches, mandated in CBME since 2019, that can achieve the goal of lifelong learning and also helps to reduce the number of demotivated medical graduates, thus empowering undergraduates to achieve COs [7].

The curriculum planners advocate for the frequent use of SDL to cultivate lifelong learning skills among medical undergraduates. The SDL approach is one choice for a teaching strategy that fits with core competencies, like prescription writing and prescription auditing. As a result, SDL functions as a teaching-learning approach to acquire subject-topic knowledge as an immediate outcome and as a goal to become a lifelong learner [8]. The teacher's job is not to impart knowledge; rather, it is to facilitate learning. They ensure that the learner remains consistent with the specified learning objective [9]. NMC has allotted 10 academic hours to SDL in the subject of pharmacology. SDL is mandated within CBME in India; however, routine SDL implementation in undergraduate pharmacology is often inconsistent and not systematically aligned to COs and assessment strategies, limiting its potential to support structured competency attainment. Creating an SDL method that matches the desired outcomes is important for making SDL in pharmacology teaching more consistent, effective, and impactful by improving alignment between learner-driven activities, predefined COs, and assessment strategies within the CBME framework. A mixed-method approach was chosen to evaluate the learning thoroughly by combining objective data like scores with students' personal thoughts and experiences about the intervention. While a sequential design could explain earlier collected numbers with later qualitative data, this study used a convergent mixed-method design because both types of data were gathered at the same time and combined during analysis to better understand the results.

With this background, the present study was aimed at implementing and evaluating an outcome-based self-directed learning (OBSDL) approach for selected pharmacology competencies among second-phase (second professional year) Bachelor of Medicine and Bachelor of Surgery (MBBS) students, assessing short-term learning outcomes and student perceptions using a convergent mixed-method design. The objectives of the OBSDL approach were as follows: to implement SDL sessions that align with the curricular framework, to implement lesson plans in SDL sessions, and to obtain student perception on SDL.

## Materials and methods

Ethical approval was provided by the Institutional Ethics Committee in compliance with the Helsinki Declaration for Ethical Principles of Medical Research Involving Human Subjects (IEC/RMCH/21/2025/JAN, dated January 24, 2025). All students were informed about the nature and purpose of the study, and participation was entirely voluntary. Written informed consent was obtained from each participant. The confidentiality and anonymity of participants were not revealed. In this convergent mixed study, quantitative (pre-post scores) and qualitative (open-ended responses) data were collected during the same phase; qualitative findings were used to support and contextualize observed score changes and explore participants’ perceptions, and integration occurred at the interpretative level by using qualitative insights to complement and elaborate on the quantitative results.

Participants and sample size

Sample Size

The study involved 162 second-phase MBBS students from the 2023-2024 batch. The minimum sample size was 153, which was calculated using Cochran's formula for proportions. The formula is n = N / (1 + N * MOE²), where N is the total number of medical students, MOE is the margin of error (0.05), and n is the desired sample size [10].

The study was conducted during 10 SDL hours allotted to pharmacology. SDL is conducted in three batches thrice a week. A total of 162 students were divided randomly (lottery method) into three batches, each batch having 54 students. All faculty members of pharmacology were informed about the nature and purpose of the study. Verbal consent was obtained from all the faculty. As a part of the student development program conducted in the pharmacology department, all students were aware of the necessary SDL skills. Topics such as principles of adult learning, group dynamics, search strategies, search engines, medical subject headings (MeSH) words, Boolean operators, and the basics of communication skills were taught to students as soon as they entered phase 2 of MBBS in pharmacology.

The steps for the intervention are as follows: step 1, selection of competency and development of module; step 2, development and validation of lesson plan; step 3, actual conduct of the session; step 4, student perception; and step 5, end-of-module assessment.

Step 1: Selection of Competencies and Development of Module

Nominal group testing was used to select competencies for SDL. Each student was asked to give their suggested list of four topics for the SDL session. The students were free to discuss with their colleagues to choose the topics. All the participants were asked to proceed to rank the topics according to priority as 1st, 2nd, 3rd, and 4th. Participants discussed the rationale for their choices of topic. This facilitated the understanding and identification of diverse ideas and approaches by each participant. The rank for each topic received was summed up, and the topic with the highest total ranking was selected as the final decision for the development of the module. The module was drafted by the first author (professor and head, also Medical Education Unit (MEU) coordinator, Advanced Course in Medical Education (ACME), and a Foundation for Advancement of International Medical Education and Research (FAIMER) fellow). The draft module was shared with the other three senior faculties of pharmacology (professor rank, all trained in medical education technologies (MET) and resource faculties for the same) for review. The other three experts (all trained in MET) were from different colleges. Finally, the module was submitted to the curricular committee for final approval. The module consists of a lesson plan, specific learning objectives (SLOs), and teaching, learning, and assessment methods for each chosen competency. Resources for learning were also identified. A total of 15 pretest and 15 posttest multiple-choice questions (MCQs) were finalized for the 1st, 2nd, and 4th competencies, and three objective structured practical examination (OSPE) stations with checklists were designed for the 3rd competency. The following four competencies were chosen: (1) skeletal muscle relaxants (SMR), (2) pharmacotherapy of glaucoma (PG), (3) management of common poisoning (MCP), and (4) communication skills (CS).

Step 2: Development and Validation of the Lesson Plan

The lesson plan was developed based on the CBME guidelines and “The Kemp's Instructional Design Model” [4,11]. A lesson plan is a detailed outline developed to structure an educational intervention, which includes the name of the competency, domain, and level of learning; the number of learners; the year of instruction; SLOs; resources required to learn; the teaching method chosen; the duration of the session; the breakup of the session; teaching aids required; infrastructure required; prior student preparation required or not; the assessment method chosen; or any other relevant activity. Lesson plans varied from topic to topic with different teaching strategies. Lesson plans were shared with students one week before the conduct of the SDL activity. The validation of the lesson plan was initially done by nominal testing followed by the Delphi method, taking the consensus from 10 experts. Seven content experts were within the department of pharmacology, and three were from other colleges. All experts were trained in MET. After four Delphi rounds, the lesson plan for each SDL session was finalized. Kendall's coefficient of concordance test (W) and Fleiss' kappa were analyzed to indicate the consensus reached by experts [11].

Step 3: Actual Conduct of the Session (Two Contact Sessions and an Intersession Period of One-Week Gap Between Two Contact Sessions)

1st contact session: A total of 54 students in each batch were divided into seven groups with one facilitator allotted to each group. A pretest was conducted. Each MCQ had three distractors and one correct option. There was no negative marking for incorrect responses. A brief instruction was given to the participants about what was expected of them. These students were provided with the SLOs and guidance for reference books to refer to. They were asked to come prepared for the next contact session after one week of intersession period.

Intersession period of one week duration: A WhatsApp group of students with a facilitator was created for each group. This period was utilized to clarify any doubts. Facilitators helped the students identify more resources. Students were allowed to meet their facilitators personally. This was followed by a second contact session.

Second contact session: It included more personalized teaching and learning, better use of class time, and flexible technology and allowed students to take more responsibility for their own learning. The students were encouraged to work and discuss with the group members, with a faculty member acting as facilitator. Teaching approaches and assessment methods varied with the competencies. The flipped classroom for SMR, the Jigsaw method for PG, role play for CS, and self-paced learning for MCP were chosen as teaching-learning methods for SDL [8]. Students' perceptions were obtained. Assessment was conducted in the form of MCQs (according to Bloom's level of learning) and OSPE (showing which level). The pretest was conducted before the start of each SDL session, and the posttest was conducted after the completion of the SDL module.

Step 4: Student Perception

Student perspective on each SDL session was assessed by responses obtained after asking structured open-ended questions. Perceptions were obtained in groups after every 2nd contact session for SMR, PG, and CS. For MCP, perception was obtained after the 1st contact session (no 2nd contact session for MCP). Each group had one facilitator asking structured open-ended questions and noting the responses verbatim. “Please reflect on the teaching methodology used in the SDL sessions,” and “mention advantages and challenges with the method used” were the questions asked. At the end of the SDL module, an overall reflection about OBSDL was obtained. Thematic analysis was performed by a two-person analysis team who had prior experience in qualitative research [12]. Responses were analyzed by reading all the written verbatim. Frequently used representative comment words or phrases were identified that may indicate key findings. The key findings were given independent primary codes by the two coders. The primary codes with similar concepts were merged, and final codes were decided in order to find themes or patterns. The codes generated and representative comments were then reviewed by all the authors to ensure credibility. Reliability and validity of thematic analysis were done by calculating intercoder reliability statistics (Cohen’s kappa) to assess the level of agreement between two coders.

Step 5: End-of-Module Assessment

A posttest having 15 MCQs of varied levels of Bloom's taxonomy was given for SMR, PG, and MCP, and three OSPE stations were designed for CS.

Statistical analysis

Statistical analysis was performed using Jamovi (version 2.7). Data normality was assessed using the Shapiro-Wilk test. Validity of the lesson plans was evaluated using Kendall’s coefficient of concordance and Fleiss’ kappa coefficient. To compare pre-test scores in different skills, we used repeated-measures analysis of variance (ANOVA) with a Greenhouse-Geisser adjustment, and then pre- and posttest scores were compared using the paired t-test. Qualitative responses were analyzed using thematic analysis, and interrater agreement between two coders was assessed using Cohen’s kappa.

## Results

Of the 162 students in the cohort, 156 were present throughout all SDL sessions and were included in the analysis. The performance of the assessments was tabulated in a spreadsheet. The normality of the data was checked using the Shapiro-Wilk test, and it was normally distributed (p > 0.05). Data analysis was done using Jamovi (version 2.7). Tables 1-4 represent lesson plans. Lesson plans for two contact sessions were prepared for SMR, PG, and CS, and one contact session for MCP, respectively. Each lesson plan has a different teaching-learning strategy and was provided with all necessary instructions.

**Table 1:** Lesson plan-skeletal muscle relaxants K: knows; KH: knows how; S: shows; SH: shows how; SLOs: specific learning objectives; SGD: small group discussion; MCQs: multiple-choice questions; MBBS: Bachelor of Medicine and Bachelor of Surgery; -: not applicable; †: domain levels of learning; ‡: breakup of session

Parameter	1st contact session	2nd contact session
Name of competency	Skeletal muscle relaxants	Skeletal muscle relaxants
Knowledge (K)^†^	Yes	Yes
Knowledge-how (KH)^†^	Yes	Yes
Show (S)^†^	No	No
Show how (SH)^†^	No	No
No. of learners	54	54
Year of instruction	2nd phase MBBS	2nd phase MBBS
SLOs and guidance for resources	Yes	Yes
Teaching strategy chosen	SGD	Flipped classroom
Duration of session (min)	60	120
Introduction^‡^	Yes	–
Formation of groups and briefing^‡^	Yes	–
Small group discussion (SGD)^‡^	–	Yes
Case-based discussion^‡^	–	Yes
Quiz^‡^	–	Yes
Clarification of doubts^‡^	Yes	Yes
Student perception^‡^	–	Yes
Attendance^‡^	Yes	Yes
Teaching aid – Audio visual aids	Yes	Yes
Reading material provided	Yes	Yes
Infrastructure required	Pharmacology lab with seating arrangement	Pharmacology lab with seating arrangement
Student preparation for 2nd session	Yes	No
Assessment method chosen	5 MCQs (pretest)	Posttest (5 MCQs) will be conducted after completing all modules of SDL
Perception	–	Yes

**Table 2:** Lesson plan-pharmacotherapy of glaucoma K: knows; KH: knows how; S: shows; SH: shows how; SLOs: specific learning objectives; SGD: small group discussion; MCQs: multiple-choice questions; MBBS: Bachelor of Medicine and Bachelor of Surgery; -: not  †: domain levels of learning; ‡: breakup of session

Parameter	1st contact session	2nd contact session
Name of the competency	Pharmacotherapy of glaucoma	Pharmacotherapy of glaucoma
Knowledge (K)^†^	Yes	Yes
Knowledge-how (KH)^†^	Yes	Yes
Show (S)^†^	No	No
Show-how (SH)^†^	No	No
No. of learners	54	54
Year of instruction	2nd phase MBBS	2nd phase MBBS
SLOs and guidance for resources	Yes	Yes
Teaching strategy chosen	SGD	Jigsaw
Duration of session (min)	60	120
Pretest (MCQs/OSPE)^‡^	Yes	No
Formation of groups and briefing^‡^	Yes	No
Jigsaw/small group activity^‡^	No	Yes
Group discussion and clarifying doubts^‡^	Yes	No
Group presentations^‡^	No	Yes
Student perception^‡^	No	Yes
Attendance^‡^	Yes	Yes
Teaching aid–audiovisual aids	Yes	Yes
Reading material provided	No	Yes
Infrastructure required	Pharmacology lab with seating arrangement	Pharmacology lab with seating arrangement
Student preparation for 2nd session	Yes	No
Assessment method chosen	5 MCQs (pretest)	Posttest (5 MCQs) after completion of SDL modules
Perception	No	Yes

**Table 3:** Lesson plan-communication skills K: knows; KH: knows how; S: shows; SH: shows how; SLOs: specific learning objectives; SGD: small group discussion; MCQs: multiple-choice questions; OSPE: objective structured practical examination; SDL: self-directed learning; †: domain levels of learning; ‡: breakup of session

Parameter	1st contact session	2nd contact session
Name of the competency	Communication skills	Communication skills
Knowledge (K)^†^	Yes	Yes
Knowledge-how (KH)^†^	Yes	Yes
Show (S)^†^	No	No
Show-how (SH)^†^	Yes	Yes
No. of learners	54	54
Year of instruction	2nd phase	2nd phase
SLOs and guidance on resources	Yes	Yes
Teaching strategy chosen	SGD	Role play
Duration of session (min)	120	120
Pretest^‡^	Yes	No
Formation of groups and briefing^‡^	Yes	No
Allotment of scenarios and group discussion^‡^	Yes	No
Explaining principles of communication^‡^	No	Yes
Demonstration role play by faculty^‡^	No	Yes
Role plays by student groups^‡^	No	Yes
Brainstorming and feedback^‡^	No	Yes
Student perception^‡^	No	Yes
Attendance^‡^	Yes	Yes
Teaching aid-audiovisual aids	Yes	Yes
Reading material provided	No	No
Infrastructure required	Pharmacology lab with seating arrangement	Pharmacology lab with seating arrangement
Student preparation for 2nd session	Yes	No
Assessment method chosen	Pretest OSPE with checklist	Posttest OSPE with checklist after SDL
Perception	No	Yes

**Table 4:** Lesson plan-management of common poisoning K: knows; KH: knows how; S: shows; SH: shows how; SLOs: specific learning objectives; SGD: small group discussion; SDL: self-directed learning; MCQs: multiple-choice questions; MBBS: Bachelor of Medicine and Bachelor of Surgery; OSPE: objective structured practical examination; †: domain levels of learning; ‡: breakup of session

Parameter	Single contact session
Name of the competency	Management of common poisoning
Knowledge (K)^†^	Yes
Knowledge-how (KH)^†^	Yes
Show (S)^†^	No
Show-how (SH)^†^	No
No. of learners	54
Year of instruction	2nd phase MBBS
SLOs and guidance on resources	No
Teaching strategy chosen	Interactive large group teaching followed by SGD
Duration of session (min)	60
Pretest (MCQs/OSPE)^‡^	Yes
Introductory session on self-paced learning^‡^	Yes
Student-led group discussion using devices^‡^	Yes
Attendance^‡^	Yes
Teaching aid–audiovisual aids	Yes
Reading material provided	No
Infrastructure required	Pharmacology lab with seating arrangement
Student preparation (posttest)	Yes
Assessment method chosen	5 MCQs
Perception	No

Table 5 represents the validity of the lesson plan. The findings from Kendall’s coefficient of concordance indicate a progressive improvement in expert agreement across the four Delphi rounds. Finally, the level of agreement was moderate across all lesson plans, reflecting a higher level of concordance among the panel. Fleiss' kappa coefficient (K) also indicates moderate agreement. 

**Table 5:** Validity of all lesson plans SMR: skeletal muscle relaxants; PG: pharmacotherapy of glaucoma; CS: communication skills; MCP: management of common poisoning; W: Kendall’s coefficient of concordance; K: Fleiss’ kappa coefficient; z: standard normal deviate (test statistic for Fleiss’ κ); χ²: chi-square statistic (significance test for Kendall’s W); p: probability value

Delphi round	Kendall’s coefficient of concordance (W)	χ 2	p-value	Fliess kappa coefficient (K)	Z	p-value
Lesson plan 1 (SMR)	W = 0.443	35.88	<0.001	K = 0.370	7.44	<0.001
Lesson plan 2 (PG)	W = 0.450	36.45	<0.001	K = 0.378	7.49	<0.001
Lesson plan 3 (CS)	W = 0.445	36.04	<0.001	K = 0.372	7.60	<0.001
Lesson plan 4 (MCP)	W = 0.532	43.09	<0.001	K = 0.435	8.74	<0.001

Assessment questions for pretest and posttest

The questions reflected varied levels of Bloom's taxonomy and Miller's pyramid. Repeated measures ANOVA with Greenhouse-Geisser, followed by post hoc analysis using Bonferroni, revealed that mean pretest scores differed statistically significantly between different competencies (p < 0.001). The mean pretest score was statistically significantly increased from SMR to glaucoma (p < 0.001), from SMR to poisoning (p < 0.001), from glaucoma to CS (p < 0.001), from glaucoma to poisoning (p < 0.001), and from CS to management of poisoning (p < 0.001).

Table 6 represents the mean scores of the pretest and posttest obtained by students. The difference between the performances of students before and after going through the above teaching modalities was assessed using a paired t-test. Results are shown as mean ± standard deviation. The p-value of data entry was calculated using Jamovi (version 2.7). A p-value of less than 0.05 was deemed statistically significant. All learning methods were effective, as indicated by the significant increase in posttest scores compared to pretest scores.

**Table 6:** Scores before and after SDL sessions for each competency SMR: skeletal muscle relaxants; PG: pharmacotherapy of glaucoma; CS: communication skills; MCP: management of common poisoning; *t*: student’s t-statistic; p: probability value

Competency	Pretest	Posttest	t	p-value
SMR	0.413 ± 0.404	1.53 ± 0.355	-25.92	<0.001
PG	0.545 ± 0.407	1.48 ± 0.414	-19.85	<0.001
CS	0.356 ± 0.412	1.4 ± 0.411	-19.95	<0.001
MCP	0.857 ± 0.492	1.52 ± 0.401	-22.41	<0.001

**Figure 1:**
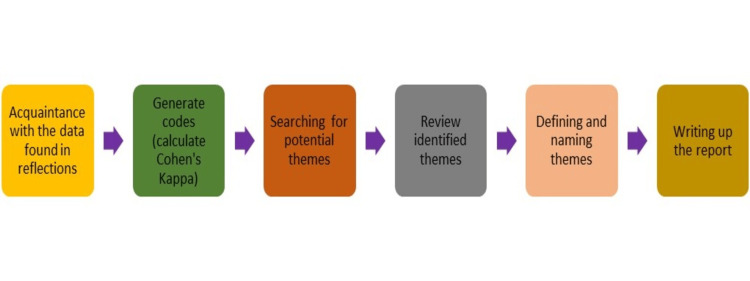
Process of thematic analysis

Figure 1 represents the process of thematic analysis. The researcher followed a 16-item checklist to ensure complete thematic analysis [12].

Table 7 represents thematic analysis. Thematic analysis initially revealed 61 codes of all SDL activities, including lesson plans, flipped classrooms, jigsaw, role play, self-paced learning, intersession periods, and overall OBSDL. Five primary codes were discarded following a review by two coders due to redundancy or insufficient iteration over the data. Cohen's kappa was 0.672, which indicated substantial agreement between the two raters.

**Table 7:** Thematic analysis SDL: self-directed learning; OBSDL: outcome-based self-directed learning

Parameter	Codes	Themes								Sample Representative comments
Lesson plan	Introduces important objectives	Self-learning skills				Metacognition				“Lesson plan for SDL was very useful for me as it gives opportunity to learn in a structured way”
	Organize learning									
	Preparation for classes									
	Self-reading									
	Decreased class boredom									
Flipped classroom	Self-learning	Self-learning skills		Metacognition		Collaborative learning		Application		“It is easier to understand that the topic is read at least twice then discussion in class makes it more informative for us”
	Effective learning									
	Better comprehension									
	Peer learning									
	Increased participation									
	Application of knowledge									
Jigsaw	Engagement	Self-learning skills	Collaboration	Social constructivism	Heutagogy	Metacognition		Group dynamics		“Firstly nervous but I could easily understand to the Jigsaw method after teacher has explained”
	Teamwork									
	Active learning									
	Joyful learning									
	Communication									
	Uneven participation									
	Distractions									
	Time management									
	Interesting way of learning									
	Critical thinking									
Role play	Effective	Collaboration		Experiential Learning		Andragogy		Behaviorism		“Atmosphere during role play was non-threatening and non-judgmental”
	Interesting									
	Peer learning									
	Joyful learning									
	Theoretical knowledge									
	Nonjudgemental									
	Nonthreatening atmosphere									
	Casual approach									
Self-paced learning	Self-control	Autodidactism		Heutagogy			Behaviorism			“I am happy to study by my own"
	Self-concept									
	Self-discipline									
	Proactive									
	Less complicated topic									
Intersession period	Innovative	Experiential learning		Constructivism		Mentoring		Autodidactism		“Faculty involvement and guidance during this period was very helpful”
	Understanding concepts									
	Curiosity									
	Self-control									
	Expert guidance									
	Increased bonding									
	Group learning									
	Time management									
	Active participation									
Overall experience with OBSDL	Active learning	Self-learning skills	Metacognition	Autonomy	Collaboration	Lifelong learning skills	Application	Mentoring	Effective learning	“SDL can be a supplemented way of teaching for few topics”
	Time management									
	Problem-solving skills									
	Delightful peer learning and teaching									
	Long-term retention of information									
	Cooperative faculties									
	Collaborative effort									
	Application of Knowledge									
	Assessment drives learning									
	Critical thinking									
	Self-management									
	Self-monitoring									
	Motivation									
	Mentor									

## Discussion

CBME emphasizes that Indian medical graduates function as lifelong learners and have a continuous commitment to improve knowledge and skills [4]. Teaching pharmacology in this context is an art that transfers knowledge from instructor to student using a competent teaching-learning exchange process to impart required knowledge and skills. A combination of teaching methodologies should be practiced and complemented to facilitate learning among students [13].

SDL has gained importance in the medical curriculum since its formal inception in CBME, besides traditional teaching. Fixed hours of SDL have been allotted to different subjects. Numerous studies have compared SDL to traditional lectures, with some of these studies demonstrating that self-learning groups achieved better results than groups who received lectures, while other studies have found no difference between SDL and traditional classroom teaching [14]. The researchers in this study did not aim to compare SDL to traditional lectures, but rather framed an outline to achieve the outcome in SDL at its full potential. Since both the teaching methods are beneficial, suited in different contexts, and have merits and demerits of their own. They should be utilized complementarily and not contradictively. For medical students, SDL is a beneficial and effective learning tool. Undergraduate students are getting SDL education in a variety of modalities, and multiple studies have shown how successful SDL is at improving student readiness and interest. The several SDL modalities included in different medical curricula work well if the objectives are achievable and feasible, allowing students to use SDL methods under conditions where independent study is required [14]. While SDL is challenging to implement, the researchers in this study have tailored the strategy for SDL according to the requirements of the competency and SLOs to enhance the applicability and impact in achieving COs in the discipline of pharmacology. In order to achieve COs, curriculum alignment is deemed essential. A proper framework in the form of a lesson plan for SDL had been developed and implemented. This strategy is supported by Du Toit-Brits et al., who stated that faculties must also be able to effectively create lesson plans, identify learning objectives, and develop inspirational methods in order to gain maximum benefit from SDL [15]. Many educational concepts and methodologies have emphasized the value of organized lesson planning, especially in the context of outcome-based education [16].

On thematic analysis, it was observed that metacognition and self-learning skills were promoted by the lesson plan. Learners were more focused and confident in class. Many studies have concurred with this observation that, when students are aware of the intended objectives of the lesson, they are more likely to plan, track, and assess their learning outcomes, improving academic performance, motivation, and satisfaction, as well as encouraging cognitive processing [17].

A successful teaching approach should be designed to enable learners to assimilate and apply the knowledge they have learned. The emphasis should not only be on the content but also on the process of delivering the material as well [18].

The researchers in this study have employed diverse teaching strategies based on the nature of the competencies to be imparted through SDL. Flipped classroom, Jigsaw, role play, and self-paced learning methods were used to teach SMRs, PG, CS, and MCP, respectively. With the flipped classroom model, the majority of the teacher-centered education takes place outside of the classroom, allowing students to explore topics independently and use classroom time to clear up any confusion and engage in more student-centered learning activities [11]. Numerous student-centric learning activities could be conducted in class during flipped teaching, like problem-solving exercises, case discussions, presentations, and group discussions [19]. Similarly, in our study, case-based discussions in small groups, quizzes, and solving doubts were conducted during classroom learning in a flipped classroom. Numerous research corroborated our thematic analysis findings that flipped classrooms are a useful strategy for improving student metacognition, collaborative learning, increasing engagement in class, increasing the likelihood that students will participate in interactive sessions, lessening anxiety about exams, and achieving better results [20].

Our study's Jigsaw results are consistent with a study by Wani et al., who found that both SDL and Jigsaw sessions improved posttest scores, demonstrating the value of active learning techniques in raising students' academic achievement. Differing with respect to the design of this study, Wani et al. had compared Jigsaw with SDL; however, in this study, Jigsaw was utilized as a teaching tool for SDL during class because the constructivist theory of SDL and the adult learning principle serve as the foundation for Jigsaw, an active learning technique. Wani et al. also concurred that the best results might be obtained from an integrated paradigm that uses SDL to encourage autonomous preparation first, then Jigsaw sessions to reinforce knowledge through peer teaching [10]. Our findings are also congruent with those of a few other studies that aimed to investigate the effects of Jigsaw [21]. Thematic analysis revealed that collaboration and the heutagogy approach, like learner autonomy, capacity, and capability is essential to the success of the Jigsaw technique, which is further validated by other research that the Jigsaw method encourages a sense of responsibility for both individual and group members' collective learning [21,22]. Each student and group member learns independently in this method and enhances their understanding, communication, teamwork, and interest in uninteresting topics, which supports its use as a teaching strategy in SDL [23]. Despite numerous benefits, the Jigsaw method also offered several limitations as embedded in social constructivism. A few representative comments, like “My communication ability is not good,” “My few friends are not willing to participate actively,” depicted the challenges of this teaching tool. Similar observations were mentioned by Wani et al. in their study [10].

Another teaching tool for SDL used in our study was role play. Role play is suited for teaching soft skills to students and professionals [24]. Similarly, the researchers in the current study also employed this technique in imparting good CS. Collaboration, experiential learning, andragogy, and behaviorism were key themes identified. Role play, when properly planned and executed, has favorable aspects of joyful learning and significantly increases the cognitive, emotional, and psychomotor domains [24]. In our study, a brief overview of communication was imparted to students that ensured everyone had a similar understanding of the content, and role play exercises were carefully tailored to meet SLOs, which is well-supported by other studies that lectures combined with practical components had the most significant positive impact on the improvement of CS [25]. This experiential learning approach not only enhances comprehension but also fosters a genuine interest in this subject, thereby enhancing student engagement throughout the learning journey and demonstrating a change in behavior, which is also observed in the other studies [26]. Thus, role play should be a priority in the current medical curriculum to promote a learning attitude and CS [27].

Self-paced learning was also one of the SDL teaching approaches used in this study. Autodidactism, heutagogy, and behaviorism were themes that emerged, encouraging learners to learn independently. A few representative comments of students, like “It’s easy to read easy and less complicated topics by myself” and “self-study of complicated topic is somewhat difficult,” revealed that this form of learning is preferred for less complicated topics, which require lower-order thinking skills. This observation is well-supported by the study of Babu et al. [18]. This reveals that the dependence on self-paced learning to deliver information cannot be advisable. This also depends on the topics involved because some of them require the application of knowledge for clinical and practical purposes. Certain topics needed an additional effort and guidance from facilitators as well [28]. However, self-paced learning transforms students into adult learners, which is a central component of SDL [18].

In this study, the intersession period between two contact sessions was one week, which was utilized for self-study to discuss any queries related to the topic with the facilitators. The facilitators were available both offline (during college hours) and on WhatsApp (online). Similar guidelines are mentioned by Charokar et al. and Anshu et al. [8,9]. The one-week duration of the intersession period was well-supported by Charokar et al. that the duration of the intersession period should not be more than 14 days, as it decreases the focus about SDL topic [8]. Experiential learning, constructivism, mentoring, and autodidactism were themes identified, all of which are necessary skills for SDL, which students perceived throughout the SDL activities.

Assessment was conducted to analyze the achievement of outcomes. Pretest at the beginning of each SDL session and posttests at the end of all SDL sessions were analyzed. The pretest MCQ results indicated that there were significant statistical differences (p < 0.001) in the baseline knowledge between all four competencies, which indicated that the students were already self-directed for a few topics, and they had read the topic before it was formally taught through SDL, and their readiness for SDL is high. This could have been possible because of the student development program, which is conducted in the department of pharmacology as a routine after the commencement of the second year MBBS, in which students are sensitized about various strategies of SDL, which attributed to higher initial motivation to self-study and perform better. The improvement in posttest scores following SDL sessions confirms the effectiveness of active learning strategies in enhancing students' academic performance and achieving COs. This finding aligns with existing literature, which emphasizes the superiority of interactive, student-centered approaches over didactic lectures in promoting knowledge acquisition and retention among medical undergraduates, which is helpful in achieving COs [29].

To achieve SDL outcomes, trained faculties are the keystone, for which a robust training program for teachers and students are must [8]. Akin to this, all researchers in this study were trained in MET and rigorously conducted student and faculty development programs regarding SDL in the institute.

Overall, in OBSDL, the themes of self-learning skills receive the greatest attention from students, followed by metacognition, autonomy, collaboration, lifelong learning skills, application, and mentoring. All of these are essential SDL skills that learners acquire during SDL, and the role of the facilitator is well-perceived with the theme mentoring. Lesson plans for different topics, SLOs, guidance to access the right resources, and post-SDL assessment were very beneficial in achieving outcomes. No singular, universal method is applicable to all circumstances and learners. This study has important limitations. First, the evaluation used a single-group pre-post design without a control or comparator group; therefore, the observed score improvements cannot be attributed solely to the OBSDL approach, as alternative explanations such as maturation, concurrent learning activities, testing effects, and instructor influence may have contributed. Second, the intervention was implemented in a single institution and within one academic cohort, which limits external validity and generalizability to other settings with different curricula, faculty expertise, or learner profiles. Third, outcomes were assessed over a short period and focused primarily on immediate performance gains; longer-term retention, transfer to clinical application, and sustained SDL behaviors were not measured. Fourth, the effect sizes were not provided, making it hard to understand how important the improvements were in real-life terms, not just in statistics. Finally, the qualitative component relied on open-ended student responses and may be subject to response and social desirability bias, and facilitator involvement in implementation could introduce expectancy effects despite standardization efforts. Future studies should involve multiple centers with comparison groups, report effect sizes to measure the educational impact, and include long-term follow-ups to better determine how effective and sustainable the results are and how widely they can be applied. The implementation of many strategies in varied combinations, both in the classroom and online, benefits learners in achieving COs. The main goal of this study is to motivate health professionals to create outcome-focused strategies in their organizations to effectively support SDL in line with CBME.

## Conclusions

The current study shows that using OBSDL methods in undergraduate pharmacology is possible and leads to better short-term learning results in our environment. The study results also show that students made significant short-term score improvements in specific pharmacology skills when using an outcome-aligned OBSDL approach, along with positive perceptions reflected in the qualitative themes. These study results support the idea of using OBSDL as part of a hands-on learning approach for teaching pharmacology that fits with the CBME framework. However, broader applicability should be interpreted cautiously because this was a single-center, single-department study, and the evaluation focused on short-term outcomes without a control/comparator group. Subsequent research ought to investigate long-term retention and practical application, utilize comparator designs across various institutions, and integrate structured satisfaction metrics, such as Likert-scale-based feedback from students and faculty, to enhance the evaluation of acceptability and scalability.

## Appendices

**Table 8:** Specific learning objectives of competencies chosen SMR: skeletal muscle relaxants; PG: pharmacotherapy of glaucoma; CS: communication skills; MCP: management of common poisoning

Competency 1: SMR	
The MBBS phase 2 student must be able to:	
1. Classify skeletal muscle relaxants.	
2. Explain the mechanism of action of depolarizing NM blockers	
3. Explain the mechanism of action of nondepolarizing NM blockers	
4. Discuss the differences between depolarizing and nondepolarizing skeletal muscle relaxants	
5. Discuss the therapeutic uses and adverse effects of skeletal muscle relaxants	
6. Explain centrally acting skeletal muscle relaxants (spasmolytics)	
7. Enumerate drugs causing malignant hyperthermia	
8. Discuss the rationale for the use of dantrolene in the treatment of malignant hyperthermia	
9. Discuss succinylcholine apnea and its management	
Competency 2: PG	
The MBBS phase 2 student must be able to:	
1. Define glaucoma	
2. Discuss the pathophysiology of glaucoma	
3. Discuss types of glaucoma	
4. Discuss clinical features of glaucoma	
5. Classify drugs used in glaucoma	
6. Discuss the mechanism of action of drugs for glaucoma	
7. Discuss the rationale for the use of individual drugs for glaucoma	
8. Discuss side effects	
9. Discuss contraindications of drugs	
10. Elaborate treatment plan for a given case scenario	
Competency 3: CS	
The MBBS phase 2 student must be able to:	
1. Define active listening	
2. Discuss components of active listening	
3. Discuss principles of communication	
4. Discuss verbal and nonverbal communication	
5. Communicate the prescription to the standardized patient in front of the observer using the 5’R format	
6. Communicate with the standardized patient regarding optimal use of a) drug therapy, b) devices, and c) storage of medicines	
7. Explain the given medication regimen and possible adverse effects of the drugs to the standardized patient	
Competency 4: MCP	
The MBBS phase 2 student must be able to:	
1. Enumerate drugs causing poisoning	
2. Explain general measures in management of poisoning	
3. Discuss specific measures, including antidotes depending on the nature of the poison	

**Table 9:** Assessment questions SMR: skeletal muscle relaxants; Ach: acetylcholine; neuromuscular; PG: pharmacotherapy of glaucoma; MCP: management of common poisoning; CS: communication skills; OSPE: objective structured practical examination

Pretest questions	Posttest questions	Bloom's taxonomy
Competency 1: SMR		
1. Which of the following is a depolarizing neuromuscular blocker?	1. Continued exposure of muscle endplates to succinylcholine results in their:	Remember
a. Vancuronium	a. Conversion to ion channel	
b. Cisatracurium	b. Enhanced sensitivity to Ach	
c. Succinylcholine	c. Regeneration of Ach receptors	
d. Tubocurarine	d. Repolarization	
2. Phase 1 block is:	2. Anticholinesterase agents are widely used to reverse the neuromuscular blockade caused by which of the following?	Understand
a. Persistent endplate repolarization	a. Succinylcholine	
b. Persistent endplate desensitization	b. Tubocurarine	
c. Persistent endplate downregulation	c. Hemicholinium	
d. Persistent endplate depolarization	d. Acetylcholine	
3. Why is atropine often administered with anticholinesterases to reverse the effects of NM blockade?	3. Succinylcholine causes cardiac arrest when administered to burn patients because it causes:	Apply
a. To prevent bradycardia	a. Hypokalemia	
b. To prevent tachycardia	b. Hypocalcemia	
c. To reverse NM blockade	c. Hyperkalemia	
d. To augment the effect of anticholinesterases	d. Hypercalcemia	
4. Which is the most appropriate answer?	4. What is the most appropriate answer?	Analyze
a. Fasciculations are the early feature of both types of NM blockers	a. Phase 1 and Phase 2 blocks are caused by tubocurarine, and the effect is reversed by neostigmine	
b. Flaccid paralysis is the early feature of both types of NM blockers	b. Succinylcholine causes phase 1 block followed by phase 2 block, and neostigmine reverses the effect of succinylcholine	
c. Anticholinesterases reverse the effect of both types of NM blockers.	c. Phase 1 and Phase 2 block are caused by tubocurarine, and the effect is NOT reversed by neostigmine	
d. The activity of Ach is blocked by both types of NM blockers	d. Succinylcholine causes a phase 1 block followed by a phase 2 block, and neostigmine does not reverse the effect of succinylcholine	
5. A 32-year-old male undergoes surgery. After that, he complains of muscle aches. Which of the following is the most correct answer?	5. A SMR was administered during a surgical procedure. The patient immediately becomes hypotensive and breathless. Which is the most correct answer?	Evaluate
a. A nondepolarizing NM blocker was administered	a. The drug is a depolarizing NM blocker	
b. The drug also commonly causes hypokalemia	b. The drug undergoes spontaneous degradation (Hofmann elimination)	
c. The drug causes persistent depolarization followed by desensitization	c. The drug causes hyperkalemia	
d. The patient is having malignant hyperthermia	d. The drug is a nondepolarizing NM blocker	
Competency 2: PG		-
1. Which of the following is a prodrug of adrenaline used topically in glaucoma?	1. Select the longer-acting ocular beta blocker:	Remember
a. Brimonidine	a. Timolol	
b. Dipivefrine	b. Betaxolol	
c. Phenylpropanolamine	c. Cartiolol	
d. Dorzolamide	d. Levobunolol	
2. Pilocarpine reduces intraocular tension in open-angle glaucoma by:	2. Beta-adrenergic blockers lower intraocular tension by:	Understand
a. Contracting sphincter pupillae	a. Downregulating adenylyl cyclase in ciliary body through reduced activation of α2 adrenoceptors	
b. Increasing tone of the ciliary muscle	b. Constricting ciliary blood vessels	
c. Reducing aqueous formation	c. Blocking adrenergic action on the trabecular meshwork	
d. Enhancing uveo-scleral outflow	d. Reducing aqueous formation unrelated to beta-adrenoceptor mediation	
3. Select the diuretic that is most effective in acute congestive glaucoma:	3. A 55-year-old female presents with a gradual loss of peripheral vision. She has no pain, and her IOP is 28 mm Hg in both eyes. What is the most likely diagnosis?	Apply
a. Indapamide	a. Primary open-angle glaucoma	
b. Amiloride	b. Acute angle closure glaucoma	
c. Mannitol	c. Secondary glaucoma	
d. Furosemide	d. Normal-tension glaucoma	
4. Betaxolol differs from timolol in that it:	4. A patient is on timolol eye drops for glaucoma but develops bradycardia and wheezing. Which alternative drug will you prescribe?	Analyze
a. Is a selective blocker	a. Pilocarpine	
b. Is more efficacious in glaucoma	b. Latanoprost	
c. Produces fewer ocular side effects	c. Dorzolamide	
d. Is longer-acting	d. Physostigmine	
5. A patient with open-angle glaucoma is poorly controlled with timolol. The next drug could be added with this:	5. A patient with acute congestive glaucoma is not responding to IV diuretics and topical miotics. What should be the next line of treatment?	
a. Pilocarpine	a. Switch to prostaglandin analogue	
b. Brimonidine	b. Increase the dosage of prescribed drugs	
c. Latanoprost	c. Perform laser peripheral iridotomy	
d. Adrenaline	d. Observe for spontaneous improvement	
Competency 4: MCP		-
1. Which of the following is a universal antidote?	1. Flumazenil is used for	Remember
a. Atropine	a. Organophosphate poisoning	
b. Desferoxamine	b. Diazepam poisoning	
c. Salted water	c. Opioid poisoning	
d. Activated charcoal	d. Paracetamol poisoning	
2. What is the role of atropine in organophosphate poisoning?	2. What is the role of N-acetylcysteine in paracetamol poisoning?	Understand
a. Blocks muscarinic receptors and provides symptomatic relief	a. It replenishes glutathione stores	
b. Blocks nicotinic receptors and provides symptomatic relief	b. Increases the excretion of paracetamol	
c. Reactivates the cholinesterase enzyme	c. Inhibits paracetamol metabolism	
d. Provides symptomatic relief by an unknown mechanism	d. Induces paracetamol metabolism	
3. A farmer, after spraying pesticides, complains of sweating, salivation, and fasciculations. The DOC in this case is	3. A patient presented within one hour of ingestion of 20 tablets of paracetamol. What is the first step in this case?	Apply
a. Atropine	a. Do gastric lavage and administer activated charcoal	
b. Naloxone	b. Administer N-acetylcysteine	
c. N-Acetylcysteine	c. Administer atropine	
d. Flumazenil	d. Forced diuresis	
4. A patient comes with the complaint of dry skin, fever, dilated pupils, and urinary retention. This could be a case of poisoning due to:	4. A patient complains of abdominal pain and vomiting. His breath was found to have a garlic odor. This could be a case of poisoning due to:	Analyze
a. Atropine	a. Lead	
b. Organophosphate	b. Iron	
c. Opioid	c. Arsenic	
d. Aspirin	d. Copper	
5. A 50-year-old male suspected of poisoning is brought to the emergency department. He complains of tinnitus, sweating, and abdominal pain. His ABG values are as follows: pH: 7.32, PaCO₂: 22 mmHg, HCO₃: 12 mEq/L. In your opinion, the above features represent which poisoning?	5. Two adult patients (A and B) are brought to the emergency department. Both are in a state of coma and respiratory depression, but with the following differences: Patient A: pin pupil. Patient B: dilated pupil and dry skin. What is the most likely cause for patient A and patient B, respectively?	Analyze
a. Organophosphate	a. Morphine poisoning; anticholinesterase poisoning	
b. Aspirin	b. Morphine poisoning; atropine poisoning	
c. Atropine	c. Anticholinesterase poisoning; morphine poisoning	
d. Opioid	d. Atropine poisoning; morphine poisoning	
Competency 3: CS		-
1. Mr. XYZ, a 55-year-old male, is diagnosed with myasthenia gravis. Communicate the diagnosis and the treatment to the patient	1. Mr. ABC, a 50-year-old male patient, is diagnosed with primary tuberculosis. Write a prescription for the patient and communicate the diagnosis and prescription to the dummy patient	OSPE stations
2. Explain the consequences of not taking the medication regularly in a patient with primary tuberculosis	2. Explain the consequences of not taking the drugs regularly in a standardized patient with leprosy	
3. Communicate regarding the use of MDI to a patient with bronchial asthma	3. Communicate with the dummy mother of a three-year-old child having diarrhea regarding the use of ORS	
